# Addressing intraarticular pathology at the time of anteverting periacetabular osteotomy for acetabular retroversion is associated with better short-term patient-reported outcomes

**DOI:** 10.1093/jhps/hnab040

**Published:** 2021-06-20

**Authors:** Joseph A Panos, Claudia N Gutierrez, Cody C Wyles, Joshua S Bingham, Kristin C Mara, Robert T Trousdale, Rafael J Sierra

**Affiliations:** 1 Mayo Clinic Alix School of Medicine, 200 1st St. SW, Rochester, MN 55905, USA; 2 Department of Orthopedic Surgery, Mayo Clinic, 200 1st St. SW, Rochester, MN 55905, USA; 3 Department of Orthopedic Surgery, Mayo Clinic, Phoenix, AZ 85054, USA; 4 Division of Biomedical Statistics and Informatics, Mayo Clinic, 200 1st St. SW, Rochester, MN 55905, USA

## Abstract

Periacetabular osteotomy (PAO) is effective in the management of developmental dysplasia of the hip and femoroacetabular impingement secondary to acetabular retroversion. During anteverting PAO for acetabular retroversion, the need for both labral treatment and femoral head–neck junction osteochondroplasty remains equivocal. Accordingly, this study evaluated patient-reported outcome measures (PROM) and reoperation rates after anteverting PAO with or without intraarticular intervention. Cases of anteverting PAO performed at a single institution between November 2009 and January 2016 were retrospectively reviewed. Patients were divided into three groups: no intervention and intraarticular intervention with arthrotomy or arthroscopy. Subsequently, patients were reclassified by the intraarticular procedure performed at surgery into major (labral repair, femoral head–neck osteochondroplasty) and minor (labral debridement, femoral/acetabular chondroplasty) groups. The cohort was 75% female, median age was 19.5 years and mean body mass index was 25.0 kg/m^2^. Preoperative to postoperative improvement was compared to minimal clinically important differences (MCID) for eight PROM. Patients receiving major interventions exceeded MCID in a greater proportion of PROM compared to minor and no intervention groups (*P* < 0.007); major or minor interventions did not increase the risk of reoperation over no intervention (*P* ≥ 0.39). Based on the current data, surgeons performing anteverting PAO for acetabular retroversion should perform arthroscopic or open labral repair and assess for impingement after the correction and perform a head–neck junction osteochondroplasty if indicated.

## INTRODUCTION

The Bernese periacetabular osteotomy (PAO) is the preferred reorientation surgery for morphologic disorders of the hemipelvis in skeletally mature patients [1]. The procedure involves four sequential osteotomies and a controlled fracture to mobilize the acetabulum, allowing for surgical adjustment of the center of rotation, and anterior–posterior or lateral coverage of the femoral head. The procedure is an appropriate method to surgically treat symptomatic developmental dysplasia of the hip (DDH) or acetabular retroversion with posterior undercoverage [1, 2].

Acetabular retroversion can present in its pure form or may be a morphologic feature present in one out of five dysplastic hips [3, 4]. If femoral anteversion is normal, both groups most often present clinically with reduced internal rotation at 90° of flexion and pain with provocative hip maneuvers. So, even in the dysplastic hip where instability is of concern, impingement of the femoral head–neck junction against the retroverted rim represents an important pathophysiological feature that leads to intraarticular damage. The repeated impaction of the anterior head–neck junction of the femur against the retroverted rim will lead to early labral damage located in the area of impingement with late separation of the labrum and acetabular cartilage [5–7]. Damage to the labrum occurs early in the process, and is in clear contradistinction to what is seen with cam impingement, where the labral damage usually occurs late, after debonding of the cartilage. Patients with retroverted sockets therefore will present commonly with pain associated with labral damage that could be worse in those hips that have combined cam morphology or femoral retroversion.

Treatment of the labrum at the time of PAO surgery done for dysplasia has been controversial. In classic dysplasia, surgeons who do not recommend treatment simply state that with correction of the dysplasia, the dysplastic labrum is mostly offloaded and could heal, or even if it does not heal, the tear could become asymptomatic [8]. This would likely not be the case with the retroverted socket. During the anteverting PAO, the anterior rim and labrum may actually be translated to a more weight-bearing position and increase its load, thus the integrity of the labrum and chondrolabral junction would seem to be much more important. Despite this theoretical concern, Zurmuhle *et al.* [2] presented a series of retroverted sockets treated with PAO without labral repair and reported better results with PAO compared to surgical hip dislocation. However, there is a paucity of literature looking at these results from the perspective of the patient using validated patient-reported outcome measures (PROMs). Intuitively, it would make the most sense that visualization and treatment of intraarticular pathologies at the time of anteverting PAO would lead to improved outcomes as the surgeon would be able to address all possible pain generators.

The aim of this study was to determine if there was a difference in PROM or reoperation rates when comparing those patients undergoing anteverting PAO with intraarticular intervention (classified as a major or minor procedure) to those without. Furthermore, differences in PROM and reoperation rates based on the extent of the intraarticular intervention performed at the time of surgery were assessed.

## METHODS

After obtaining Institutional Review Board approval (IRB No.: 17-001303), we retrospectively reviewed all patients undergoing PAO at a single tertiary referral center between November 2009 and January 2016. All patients were treated by one of two senior hip preservation surgeons (R.T.T. or R.J.S.). Candidates for PAO had symptomatic DDH or femoroacetabular impingement (FAI), defined by lateral center-edge angle (LCEA) [9] (<25° for DDH; >40° for FAI), acetabular index [10] (>10° for DDH; <0° for FAI), anterior center-edge angle (ACEA) [11] (<25° for DDH; >45° for FAI) and Tönnis Grade 0 or Grade 1 degenerative changes [12] with an age ≤50 years. We identified 212 patients (239 hips) who met these criteria. From this group, we further selected patients undergoing anteverting PAO yielding 41 patients (48 hips), which comprised the final cohort. All patients were symptomatic and complained of hip pain associated with activities. Preoperative review of pelvic radiographs identified patients with lateral undercoverage in addition to retroversion, or with pure acetabular retroversion. All patients had failed non-operative management for at least 3 months. The mean age at the time of surgery was 21.2 ± 5.6 years (range: 13.4–36.8 years) and mean body mass index (BMI) 25.0 ± 5.4 kg/m^2^ (range: 17.1–39.2 kg/m^2^) (Table I). The average preoperative range of motion for these patients was 13.5 ± 9.1° (range: 0–35°) of internal rotation at 90° of flexion and 96.8 ± 9.5° (range: 85–120°) of straight flexion (Table I). Twenty-one patients (22 hips) had combined acetabular retroversion and dysplasia (Table I).

**Table I.** Patient characteristics by surgical technique and intraarticular intervention

	Ante. PAO (*N* = 8)	Ante. PAO + arthrotomy (*N* = 23)	Ante. PAO + arthroscopy (*N* = 17)	*P*-value	No intervention (*N* = 14)	Minor (*N* = 7)	Major (*N* = 27)	*P*-value	Total (*N* = 48)
Age at surgery				0.50				0.45	
Mean (SD)	20.8 (6.1)	21.9 (6.5)	20.4 (4.0)		22.7 (6.9)	21.0 (4.5)	20.4 (5.1)		21.2 (5.6)
Median	18.5	20.0	20.1		19.6	19.8	19.3		19.5
Q1, Q3	16.9, 23.6	16.7, 26.9	17.2, 23.0		16.9, 27.9	17.8, 26.6	16.5, 23.0		16.9, 24.0
Range	(15.9–32.7)	(13.4–36.8)	(15.0–27.5)		(15.9–36.8)	(15.6–27.5)	(13.4–34.4)		(13.4–36.8)
Gender				0.78				0.65	
Female	7 (87.5%)	16 (69.6%)	13 (76.5%)		12 (85.7%)	5 (71.4%)	19 (70.4%)		36 (75.0%)
Male	1 (12.5%)	7 (30.4%)	4 (23.5%)		2 (14.3%)	2 (28.6%)	8 (29.6%)		12 (25.0%)
BMI (kg/m^2^)				0.25				0.47	
Mean (SD)	27.4 (5.6)	24.0 (4.9)	25.1 (5.9)		25.5 (5.1)	22.8 (6.1)	25.2 (5.4)		25.0 (5.4)
Median	27.0	22.8	23.1		24.2	21.3	24.4		23.2
Q1, Q3	22.6, 30.8	21.1, 25.3	21.3, 27.0		21.8, 29.0	19.2, 22.8	21.7, 27.0		21.3, 27.5
Range	(21.3–37.4)	(17.1–36.4)	(17.4–39.2)		(19.3–37.4)	(17.4–36.0)	(17.1–39.2)		(17.1–39.2)
Diagnosis				0.41^a^				0.17^a^	
DDH + retroversion	3 (37.5%)	9 (39.1%)	10 (58.8%)		8 (57.1%)	1 (14.3%)	13 (48.1%)		22 (45.8%)
Retroversion	5 (62.5%)	14 (60.9%)	7 (41.2%)		6 (42.9%)	6 (85.7%)	14 (51.9%)		26 (54.2%)
Prior surgery to affected hip				0.096				0.25	
No	6 (75.0%)	17 (73.9%)	16 (94.1%)		11 (78.6%)	4 (57.1%)	24 (88.9%)		39 (81.3%)
Yes	2 (25.0%)	6 (26.1%)	1 (5.9%)		3 (21.4%)	3 (42.9%)	3 (11.1%)		9 (18.8%)
Range of motion: flexion (deg)									
Preoperative	96.7 (10.3)	95.6 (8.9)	98.3 (10.3)	0.70	98.3 (12.2)	92.5 (2.7)	97.3 (9.4)	0.14	96.8 (9.5)
Postoperative	98.6 (5.6)	103.5 (7.0)	98.2 (6.1)	0.11	100.0 (6.1)	97.5 (4.2)	101.7 (7.5)	0.29	100.7 (6.8)
Change (Post-Pre)	2.5 (6.9)	9.4 (8.6)	1.4 (11.7)	0.086	5.0 (7.6)	5.0 (3.2)	5.4 (12.2)	0.99	5.3 (10.2)
*P*-value	0.56	0.001	0.55		0.16	0.063	0.035		
Range of motion: internal rotation (deg)									
Preoperative	13.6 (6.9)	13.5 (8.8)	13.3 (10.8)	0.99	13.5 (6.3)	10.0 (6.3)	14.3 (10.7)	0.40	13.5 (9.1)
Postoperative	27.1 (8.1)	33.3 (13.8)	27.1 (9.6)	0.27	29.4 (9.2)	28.0 (11.0)	30.2 (12.8)	0.91	29.7 (11.5)
Change (Post-Pre)	13.6 (9.0)	19.7 (12.0)	15.0 (11.8)	0.32	15.6 (8.8)	19.0 (6.5)	16.6 (13.3)	0.66	16.7 (11.4)
*P*-value	0.031	<0.001	0.001		0.008	0.063			

a Chi-square test for the difference in preoperative diagnosis (DDH + retroversion or retroversion alone) between surgical technique or intraarticular intervention groups.

For the analyses, the 48 hips were divided into three groups: no intraarticular intervention and intervention with arthrotomy or arthroscopy. A separate analysis was done by classifying hips by the extent of intraarticular intervention performed at the time of surgery into major (labral repair or femoral head–neck osteochondroplasty) and minor (labral debridement or femoral/acetabular chondroplasty) groups. A patient simultaneously receiving a major and minor intraarticular intervention was included in the major intraarticular intervention group. Patients who did not have arthrotomy or arthroscopy were included in the no intervention group. There were six additional patients (six hips) who underwent hip arthroscopy or arthrotomy in the no intervention group.

As part of a prospectively collected hip preservation registry, 12 PROM were recorded at the preoperative and most recent clinical follow-up visits, with the latter occurring at a mean of 3.2 years postoperatively (range: 0.9–5.9 years). PROM included the UCLA activity score, Harris Hip Score (HHS) [13], five subcomponents of the Hip Disability and Osteoarthritis Outcome Score (HOOS) [Pain, Activities of Daily Living (ADL), Sports and Recreation, Quality of Life], four subcomponents of the Western Ontario and McMaster Universities Questionnaire (WOMAC) (Pain, Stiffness, Physical, Total) and two subcomponents of the SF-12 Health Survey (Physical and Mental). Each score has been used previously to assess the functional outcome of patients treated with PAO for symptomatic dysplasia [14–17]. For a subset of eight PROM collected in this study, the preoperative to postoperative change was compared to the established minimal clinically important difference (MCID) reported in the literature [18].

The data are presented as counts and percentages for categorical variables or means and standard deviations for continuous variables. Retroversion index [19] and alpha angle [20] measurements were obtained from preoperative and postoperative AP pelvic and frog-leg lateral radiographs, respectively, for the 41 patients (48 hips) undergoing anteverting PAO. All postoperative AP pelvic radiographs were reviewed by a senior author to confirm appropriate correction following anteverting PAO. Comparisons of baseline characteristics and PROM (preoperative, postoperative, the change from preoperative to postoperative and the difference between the preoperative to postoperative change and MCID) were made using generalized estimating equations to account for the fact that a patient may have more than one hip included in the analysis, and that results for those hips may be correlated. Where appropriate, *post* *hoc* pairwise comparisons were conducted using the generalized estimating equations with *P*-values adjusted for multiple comparisons using the Benjamini–Hochberg false discovery rate method [21]. The proportion of PROM exceeding the MCID was compared between groups using the Fisher’s exact test. Cox proportional hazards regression with a robust variance estimator were used to assess the incidence of reoperations following anteverting PAO. Counts and the nature of postoperative complications and reoperations were confirmed in the medical record. Reoperation was defined as any additional procedure to the affected hip, not including isolated hardware removal. All analyses were performed using SAS version 9.4 (SAS Institute Inc., Cary, NC, USA) and R version 3.4.2 (R Core Team, Vienna, Austria).

## RESULTS

Forty-one patients (48 hips) are in the final cohort. Twenty patients (22 hips) underwent arthrotomy, 14 patients (16 hips) underwent arthroscopy and 5 patients (6 hips) received anteverting PAO alone. Two patients (four hips) underwent staged bilateral anteverting PAO with side-to-side differences in the surgical technique. In both cases, the left hip underwent anteverting PAO alone while the right hip received anteverting PAO + arthrotomy in one case and anteverting PAO + arthroscopy in the other. The anteverting PAO, anteverting PAO + arthrotomy and anteverting PAO + arthroscopy groups did not differ significantly by age, BMI, sex, incidence of prior surgery to the affected hip or range of motion in flexion or internal rotation (*P* ≥ 0.096) **(**Table I). There were no significant differences in preoperative Tönnis grade, LCEA, ACEA, acetabular inclination, alpha angle or retroversion index (*P* ≥ 0.25) (Table II). The preoperative to postoperative improvement exceeded the MCID for 7, 3 and 2 of 8 PROM for anteverting PAO + arthrotomy, anteverting PAO + arthroscopy and anteverting PAO alone, respectively (Table III). The proportion of PROM that surpassed the MCID was significantly greater for anteverting PAO + arthrotomy versus anteverting PAO alone (*P* = 0.04) but did not differ between anteverting PAO + arthrotomy and anteverting PAO + arthroscopy (*P* = 0.12). The distribution of PROM by diagnosis is presented in Supplementary Table SI.

**Table II.** Radiographic characteristics by surgical technique and intraarticular intervention

	Ante. PAO (*N* = 8)	Ante. PAO + arthrotomy (*N* = 23)	Ante. PAO + arthroscopy (*N* = 17)	*P*-value	No intervention (*N* = 14)	Minor (*N* = 7)	Major (*N* = 27)	*P*-value	Total (*N* = 48)
Tonnis classification				0.87				0.80	
Grade 0	6 (75.0%)	17 (73.9%)	13 (76.5%)		11 (78.6%)	5 (71.4%)	20 (74.1%)		36 (75.0%)
Grade 1	2 (25.0%)	6 (26.1%)	4 (23.5%)		3 (21.4%)	2 (28.6%)	7 (25.9%)		12 (25.0%)
Lateral center-edge angle (deg)									
Preoperative	27.0 (7.0)	25.7 (10.4)	26.4 (7.0)	0.91	26.1 (10.6)	28.7 (10.6)	25.5 (7.1)	0.74	26.1 (8.7)
Postoperative	33.6 (4.8)	32.2 (6.5)	33.1 (6.4)	0.87	33.9 (6.8)	35.4 (4.6)	31.2 (5.9)	0.26	32.7 (6.1)
Change (Post-Pre)	7.6 (5.6)	6.6 (14.2)	3.9 (4.5)	0.38	8.5 (12.7)	7.9 (14.7)	3.8 (8.4)	0.50	6.0 (10.9)
*P*-value	0.13	0.18	0.023		0.064	0.25	0.13		
Acetabular inclination (deg)									
Preoperative	6.2 (6.4)	7.8 (6.9)	10.1 (16.4)	0.70	7.9 (6.8)	5.4 (9.6)	9.3 (13.1)	0.66	8.4 (11.0)
Postoperative	−1.0 (2.2)	5.0 (6.8)	−1.7 (3.0)	0.006	4.6 (9.2)	−0.8 (5.5)	1.2 (3.1)	0.34	2.1 (6.2)
Change (Post-Pre)	−9.6 (6.4)	−3.0 (7.2)	−5.7 (3.6)	0.18	−4.8 (8.6)	−7.7 (7.2)	−4.0 (4.6)	0.54	−4.8 (6.4)
*P*-value	0.13	0.096	0.008		0.11	0.13	0.003		
Alpha angle (deg)									
Preoperative	45.6 (2.7)	52.9 (7.4)	54.5 (9.9)	0.056	46.4 (3.0)^a^	47.3 (4.4)^a^	56.4 (8.6)^b^	0.002	52.2 (8.3)
Postoperative	46.3 (2.0)	46.6 (2.3)	46.6 (2.5)	0.92	46.7 (2.0)	47.0 (3.3	46.4 (2.1)	0.84	46.6 (2.3)
Change (Post-Pre)	0.7 (1.5)^b^	−6.3 (6.7)^a^	−7.9 (10.6)^a^	0.043	0.3 (1.8)^a^	−0.3 (2.4)^a^	−10.1 (8.5)^b^	<0.001	−5.7 (8.2)
*P*-value	0.19	<0.001	0.006		0.51	0.94	<0.001		
Retroversion index									
Preoperative	35.2 (9.9)	36.1 (5.5)	36.8 (6.2)	0.89	35.6 (8.2)	35.7 (2.9)	36.6 (6.5)	0.45	36.2 (6.6)
Postoperative	7.5 (6.8)	5.9 (8.4)	8.0 (9.0)	0.80	6.2 (6.4)	4.8 (4.3)	7.7 (9.7)	0.40	6.9 (8.3)
Change (Post-Pre)	−26.5 (12.1)	−29.9 (8.1)	−28.8 (10.3)	0.78	−28.1 (10.9)	−30.9 (6.0)	−28.9 (9.7)	0.82	−28.9 (9.5)
*P*-value	0.016	<0.001	<0.001		0.001	0.016	<0.001		

a,b *Post hoc* pairwise comparisons, connecting letters report: groups that share the same letter do not differ statistically.

**Table III.** Preoperative and postoperative patient-reported outcome measures, by surgical technique performed

	Ante. PAO (*N* = 8)	Ante. PAO + arthrotomy (*N* = 23)	Ante. PAO + arthroscopy (*N* = 17)	Adjusted *P*-value^a^
UCLA score				
Preoperative	7.1 (2.3)	7.3 (2.3)	6.4 (2.9)	0.56
Postoperative	8.0 (2.0)	7.1 (2.4)	7.0 (2.2)	0.58
Change (Post-Pre)	1.0 (2.5)	−0.3 (2.8)	0.2 (2.4)	0.53
*P*-value	0.41	0.65	0.55	
Harris Hip Score				
Preoperative	61.0 (19.4)	60.5 (16.1)	56.3 (21.7)	0.81
Postoperative	89.0 (19.9)	84.2 (19.0)	78.7 (20.8)	0.51
Change (Post-Pre)	27.0 (18.1)	26.6 (21.8)	21.5 (31.7)	0.85
*P*-value	0.016	<0.001	0.023	
HOOS pain				
Preoperative	58.6 (25.7)	57.4 (19.0)	57.3 (20.1)	0.99
Postoperative	87.1 (22.5)	84.6 (21.8)	77.7 (22.3)	0.59
Change (Post-Pre)	32.9 (16.9)	29.2 (19.4)	22.1 (20.8)	0.40
*P*-value	0.031	<0.001	0.004	
MCID (10.3)^b^	0.001	<0.001	0.041	
*P*-value				
HOOS ADL				
Preoperative	72.5 (23.2)	70.0 (18.8)	68.2 (22.8)	0.90
Postoperative	89.9 (22.3)	90.6 (14.7)	87.3 (14.0)	0.81
Change (Post-Pre)	20.3 (14.7)	21.1 (18.4)	20.8 (21.5)	0.99
*P*-value	0.031	<0.001	0.006	
MCID (10.8)^b^	0.11	0.021	0.094	
*P*-value				
HOOS S&R				
Preoperative	50.9 (25.4)	44.6 (22.0)	41.3 (21.5)	0.64
Postoperative	80.5 (32.6)	75.3 (30.1)	70.1 (28.8)	0.74
Change (Post-Pre)	28.6 (30.8)	30.3 (32.4)	34.6 (30.6)	0.90
*P*-value	0.063	0.002	0.004	
MCID (12.6)^b^	0.17	0.017	0.010	
*P*-value				
HOOS QOL				
Preoperative	42.0 (16.8)	27.7 (18.4)	31.3 (21.1)	0.22
Postoperative	75.0 (30.3)	70.1 (27.2)	61.2 (25.0)	0.90
Change (Post-Pre)	33.9 (36.6)	42.4 (26.0)	34.1 (32.9)	0.65
*P*-value	0.047	<0.001	0.005	
MCID (11.2)^b^	0.10	<0.001	0.012	
*P*-value				
WOMAC pain				
Preoperative	65.0 (26.1)	63.9 (20.4)	64.3 (22.6)	0.99
Postoperative	91.9 (17.3)	87.6 (19.4)	81.8 (22.8)	0.51
Change (Post-Pre)	25.7 (18.8)	25.8 (19.5)	19.2 (22.1)	0.63
*P*-value	0.031	<0.001	0.015	
MCID (10.8)^b^	0.036	<0.001	0.17	
*P*-value				
WOMAC stiffness				
Preoperative	58.9 (24.7)	57.1 (22.2)	51.7 (27.5)	0.70
Postoperative	90.6 (14.6)	77.0 (26.1)	76.7 (18.2)	0.20
Change (Post-Pre)	30.4 (27.8)	19.7 (22.6)	25.0 (23.5)	0.67
*P*-value	0.063	0.002	0.004	
MCID (12.9)^b^	0.096	0.19	0.054	
*P*-value				
WOMAC physical				
Preoperative	72.5 (23.2)	70.0 (18.8)	68.2 (22.8)	0.90
Postoperative	91.2 (21.0)	90.6 (14.7)	88.2 (13.9)	0.87
Change (Post-Pre)	17.4 (15.4)	21.1 (18.4)	21.7 (20.9)	0.82
*P*-value	0.031	<0.001	0.003	
MCID (10.8)^b^	0.26	0.021	0.051	
*P*-value				
WOMAC total				
Preoperative	69.8 (23.2)	67.8 (18.3)	66.0 (22.2)	0.92
Postoperative	91.3 (19.5)	88.8 (15.9)	85.1 (14.8)	0.68
Change (Post-Pre)	20.2 (16.0)	21.8 (17.6)	20.8 (20.7)	0.98
*P*-value	0.031	<0.001	0.005	
MCID (10.4)^b^	0.11	0.008	0.070	
*P*-value				
SF12 physical				
Preoperative	41.2 (10.9)	37.1 (10.7)	35.4 (10.6)	0.49
Postoperative	53.3 (8.1)	49.3 (10.3)	47.3 (13.5)	0.42
Change (Post-Pre)	11.2 (3.2)	12.8 (10.8)	11.7 (14.6)	0.82
*P*-value	0.016	<0.001	0.020	
SF12 mental				
Preoperative	58.8 (8.6)	50.2 (12.6)	52.7 (10.4)	0.26
Postoperative	56.8 (5.7)	50.3 (11.3)	52.5 (9.0)	0.19
Change (Post-Pre)	−0.8 (4.1)	−0.7 (14.0)	−1.2 (12.7)	0.99
*P*-value	0.58	0.80	0.67	

a All *P*-values comparing surgical groups have been adjusted for intraarticular intervention.

b Minimal clinically important difference [18].

Twenty-two patients (27 hips) received major intraarticular interventions, 6 patients (7 hips) received minor interventions and 13 patients (14 hips) received no intraarticular intervention. The no intervention group included six patients (six hips) who received a hip arthroscopy or arthrotomy but no intervention was performed. The major, minor and no intraarticular intervention groups did not differ significantly by age, BMI, sex incidence of prior surgery to the affected hip or range of motion in flexion or internal rotation (*P* ≥ 0.25) (Table I). The alpha angle of the major intervention group was significantly greater than the minor and no intraarticular intervention group (*P* < 0.001) (Table II). There were no significant differences in any other preoperative radiographic metrics or range of motion (*P* ≥ 0.14). The preoperative to postoperative change exceeded the MCID in 8, 0 and 2 of 8 PROM for major, minor and no intervention groups, respectively (Table IV). The proportion of PROM which exceeded the MCID was greater for major interventions compared to minor or no intervention (*P* < 0.007). The distribution of PROM by diagnosis is presented in Supplementary Table SII.

**Table IV.** Preoperative and postoperative patient-reported outcome measures, by intraarticular intervention performed

	No intervention (*N* = 14)	Minor (*N* = 7)	Major (*N* = 27)	Adjusted *P*-value^a^
UCLA score				
Preoperative	7.5 (2.0)	7.7 (2.8)	6.5 (2.6)	0.34
Postoperative	7.2 (2.4)	6.4 (1.9)	7.4 (2.3)	0.61
Change (Post-Pre)	−0.3 (3.4)	−1.8 (2.2)	0.8 (1.9)	0.11
*P*-value	0.88	0.25	0.060	
Harris Hip Score				
Preoperative	60.8 (14.8)	60.1 (9.4)	58.2 (21.6)	0.89
Postoperative	81.8 (22.5)	75.9 (19.7)	85.8 (18.3)	0.52
Change (Post-Pre)	20.4 (22.1)	14.5 (16.4)	29.9 (26.8)	0.24
*P*-value	0.009	0.19	<0.001	
HOOS pain				
Preoperative	54.6 (20.3)	63.9 (14.0)	57.3 (21.4)	0.31
Postoperative	77.9 (25.9)	72.0 (24.5)	88.1 (17.8)	0.22
Change (Post-Pre)	24.8 (19.6)^b^	8.7 (13.6)^c^	34.0 (17.5)^b^	0.026
*P*-value	0.006	0.16	<0.001	
MCID (10.3)^d^	0.014	0.77	<0.001	
*P*-value				
HOOS ADL				
Preoperative	65.8 (19.1)	81.5 (11.1)	68.5 (22.6)	0.057
Postoperative	85.0 (22.2)	86.1 (11.2)	92.5 (11.9)	0.32
Change (Post-Pre)	19.4 (17.1)^b^	3.5 (11.2)^c^	27.2 (18.0)^b^	0.035
*P*-value	0.011	0.56	<0.001	
MCID (10.8)^d^	0.095	0.11	<0.001	
*P*-value				
HOOS S&R				
Preoperative	44.2 (21.6)	43.8 (20.7)	44.8 (23.5)	0.99
Postoperative	69.2 (35.7)	67.7 (28.3)	79.5 (26.4)	0.52
Change (Post-Pre)	21.9 (33.4)	22.9 (25.5)	39.3 (29.7)	0.24
*P*-value	0.071	0.13	<0.001	
MCID (12.6)^d^	0.33	0.32	<0.001	
*P*-value				
HOOS QOL				
Preoperative	36.5 (15.3)	28.6 (20.4)	29.0 (21.1)	0.30
Postoperative	67.8 (26.6)	53.1 (20.1)	72.2 (28.3)	0.25
Change (Post-Pre)	31.8 (30.4)	19.8 (28.6)	47.0 (27.8)	0.10
*P*-value	0.006	0.25	<0.001	
MCID (11.2)^d^	0.019	0.46	<0.001	
*P*-value				
WOMAC pain				
Preoperative	60.8 (20.7)	70.0 (16.3)	64.4 (23.5)	0.45
Postoperative	83.5 (23.1)	75.0 (28.3)	91.4 (14.3)	0.26
Change (Post-Pre)	20.4 (20.2)^c^	6.7 (14.0)^c^	30.2 (18.6)^b^	0.041
*P*-value	0.008	0.34	<0.001	
MCID (10.8)^d^	0.10	0.47	<0.001	
*P*-value				
WOMAC stiffness				
Preoperative	54.8 (22.0)	62.5 (22.8)	54.0 (26.0)	0.59
Postoperative	74.0 (25.7)	79.2 (18.8)	82.6 (20.6)	0.60
Change (Post-Pre)	15.6 (29.7)	12.5 (11.2)	30.7 (20.3)	0.050
*P*-value	0.14	0.13	<0.001	
MCID (12.9)^d^	0.75	0.93	<0.001	
*P*-value				
WOMAC physical				
Preoperative	65.8 (19.1)	81.5 (11.1)	68.5 (22.6)	0.057
Postoperative	86.2 (21.7)	86.1 (11.2)	92.8 (11.7)	0.32
Change (Post-Pre)	17.8 (17.3)^b^	3.5 (11.2)^c^	27.5 (17.6)^b^	0.028
*P*-value	0.011	0.56	<0.001	
MCID (10.8)^d^	0.16	0.11	<0.001	
*P*-value				
WOMAC total				
Preoperative	63.9 (18.6)	77.5 (11.9)	66.5 (22.4)	0.081
Postoperative	84.6 (21.5)	83.2 (14.1)	91.4 (12.4)	0.32
Change (Post-Pre)	18.1 (17.1)^b^	4.9 (8.2)^c^	28.1 (17.5)^b^	0.021
*P*-value	0.010	0.22	<0.001	
MCID (10.4)^d^	0.12	0.10	<0.001	
*P*-value				
SF12 physical				
Preoperative	37.2 (9.7)	37.0 (8.9)	37.1 (11.8)	0.99
Postoperative	48.9 (10.5)	43.0 (17.9)	51.5 (8.9)	0.45
Change (Post-Pre)	10.2 (9.0)	5.3 (15.9)	15.1 (10.4)	0.19
*P*-value	0.003	0.56	<0.001	
SF12 mental				
Preoperative	51.3 (14.5)	60.1 (3.1)	50.8 (10.7)	0.051
Postoperative	54.5 (7.9)	53.5 (7.9)	50.7 (11.2)	0.50
Change (Post-Pre)	3.4 (11.0)	−6.1 (8.4)	−1.9 (13.1)	0.16
*P*-value	0.73	0.16	0.54	

a All *P*-values comparing intraarticular intervention have been adjusted for surgical group.

b,c *Post hoc* pairwise comparisons, connecting letters report: groups that share the same letter do not differ statistically.

d Minimal clinically important difference [18].

Twenty additional procedures were performed in 16 patients (17 hips) following the index anteverting PAO. Fifteen patients (16 hips; 31.3%) underwent removal of painful hardware at an average of 1 year (range: 0.3–3.4 years) post-index anteverting PAO. Three patients (3 hips; 6.3%) underwent hip arthroscopy at an average of 2.2 years (range: 0.75–3.6 years) following anteverting PAO. Of these patients, two had previously received a major intraarticular intervention and underwent a second major intervention during arthroscopic reoperation. The third patient originally underwent anteverting PAO without intraarticular intervention and received arthroscopic labral debridement and cam resection at reoperation. One patient (1 hip; 2.1%) was converted to total hip arthroplasty at 2 years following anteverting PAO.

Among all patients, the cumulative incidence of reoperation-free survival (excluding hardware removal) at 1 year was 97.7% [95% confidence interval (CI) 93.3–100%] and at 2 years was 94.6% (95% CI 87.6–100%). Intraarticular interrogation in the form of anteverting PAO + arthroscopy [hazard ratio (HR) 2.15, 95% CI 0.22–21.12; *P* = 0.51] or anteverting PAO + arthrotomy (HR 0.33, 95% CI 0.02–5.30; *P* = 0.43) did not influence reoperation-free survival compared to anteverting PAO alone. Similarly, reoperation-free survival did not differ between patients receiving a major (HR 2.73, 95% CI 0.28–26.73; *P* = 0.39) or minor (HR 2.28, 95% CI 014–36.42; *P* = 0.56) intraarticular intervention relative to no intervention.

Two complications of numbness in the distribution of the lateral femoral cutaneous nerve were reported in two patients (two hips). Temporary femoral neuropathy was reported in a single patient (one hip) undergoing anteverting PAO + arthroscopy. It resolved at 6 months and at most recent follow-up, 2 years post-index anteverting PAO, the patient had normal function.

## DISCUSSION

The utility of joint interrogation at the time of PAO is a matter of controversy. In light of the acetabular correction and likelihood of the presence of labral pathology, intraarticular treatment may be the preferred strategy; however, data supporting or refuting this position is not conclusive. The purpose of this study was to determine whether surgical technique to evaluate intraarticular pathology and subsequent intraarticular interventions performed at the time of anteverting PAO influence PROM or the incidence of reoperation. The results of this study indicate that PROM of pain and function reliably improved following anteverting PAO with joint inspection using either arthrotomy or arthroscopy to a greater degree than with anteverting PAO alone. Furthermore, when evaluating the extent of the intraarticular intervention performed, consistent and clinically significant improvement in PROM with major interventions defined as labral repair or femoral head–neck junction osteochondroplasty was observed, while minor treatment or no treatment did not produce significant preoperative to postoperative clinical changes. In four categories, the magnitude of preoperative to postoperative change in PROM in the major intervention group exceeded that of the minor intervention group. Finally, at short-term follow-up, performing intraarticular intervention was not associated with an increased reoperation rate.

There are a number of limitations associated with this study. The decision to interrogate the joint with either hip arthroscopy or arthrotomy was based on surgeon expertise and preference. While this may introduce variation between study groups in the form of patient selection, we noted no differences in radiographic acetabular morphology preoperatively; however, femoral morphology was significantly different between hips that underwent major interventions compared to those that did not. Second, due to the size of our cohort and limited number of postoperative events during the period of follow-up, we were unable to conduct a multivariate analysis to examine the combined effect of surgical technique and intraarticular intervention on PROM or reoperation. We attempted to mitigate this shortcoming by examining each variable independently through separate analyses. Third, our cohort included diagnoses of DDH with retroversion and FAI secondary to acetabular retroversion. Outcomes following hip preservation surgery have been shown to differ between DDH and FAI; however, these differences have been predominantly attributed to baseline incongruities in disease severity at the time of surgery and decline by 2 years postoperatively [22]. For our cohort, neither preoperative radiographic measures of disease nor the distribution of DDH and FAI diagnoses differed between surgical technique and intraarticular intervention groups. PROMs for each diagnosis are presented in Supplementary Tables SI and SII; further subcategorization in this manner precluded comparisons between diagnoses, but underscores the need for future research comparing outcomes of anteverting PAO between DDH and FAI. Fourth, a ceiling effect in PROM could be observed. In one PROM where a differential effect was observed, preoperative scores in the minor intervention group were significantly greater than the major intervention group, possibly acting as a ceiling to limit the magnitude of postoperative improvement, explaining the differential effect observed in this instance. Finally, the short follow-up for this cohort limits our ability to determine how intraarticular intervention may change the natural history of the disease.

Anteverting PAO was first described in 1999 in a subset of 11 patients (12 hips) undergoing surgical treatment for acetabular retroversion [23]. In 2003, anteverting PAO was reported in the treatment of 22 patients (29 hips) with FAI. At an average follow-up of 2.5 years, postoperative range of motion and Merle d’Aubigné scores were significantly improved from preoperative levels [6]. The majority of this cohort (26 of 29 hips) underwent a concomitant arthrotomy with femoral head–neck offset procedure at the time of anteverting PAO. Labral pathology was present in all the hips undergoing arthrotomy and labral lesions were debrided in two hips and were not treated in the remaining 24. At 10 years of follow-up, the clinical results have been maintained, notably without conversion to total hip arthroplasty or radiographic progression of arthritis [24]. Peters *et al.* [25] reported a cohort of 54 patients (60 hips) with acetabular retroversion undergoing either anteverting PAO (30 hips) or surgical hip dislocation with osteochondroplasty (30 hips). The group receiving anteverting PAO underwent concomitant arthrotomy, with the majority of patients (87%) undergoing a major intraarticular intervention (femoral osteochondroplasty) at the time of surgery. Improvement in HHS in both groups was reported after surgery and, despite significantly lower preoperative HHS scores in patients receiving anteverting PAO, there was no difference in postoperative HHS at an average follow-up of 4 years. Parry *et al.* [26] reported outcomes of 23 patients (30 hips) with FAI due to acetabular retroversion or acetabular retroversion with dysplasia treated with anteverting PAO. The majority of cases (29 of 30 hips) received arthrotomy at the time of anteverting PAO with intraarticular procedures consisting of labral debridement in 2 cases and femoral osteochondroplasty in 11 cases. At an average follow-up of 5 years, significant improvement in HHS was reported. Further comparison of anteverting PAO to surgical hip dislocation and acetabular rim trimming in the context of acetabular retroversion has been conducted [2]. In this study, 34 of 67 hips receiving anteverting PAO additionally underwent femoral osteochondroplasty via anterior capsulotomy at the time of surgery. For the composite endpoint of conversion to total hip arthroplasty or radiographic progression of arthritis, anteverting PAO compared to surgical hip dislocation demonstrated greater survivorship at 10 years with fewer patients reporting fair or poor clinical outcomes, as judged by the Merle d’Aubigné score [2]. In clear distinction, the results of no intraarticular treatment were reported by Wyatt *et al.* [27]. In their study, the authors reported on a series of 27 patients (31 hips) undergoing minimally invasive anteverting PAO without arthrotomy or arthroscopy at a minimum follow-up of 2 years. The majority of cases in this cohort (24 hips) had failed a previous hip arthroscopy or surgical hip dislocation as index surgical treatment. Six hips required subsequent hip arthroscopy for persistent symptoms. Two hips received repeat arthroscopy with a minor intraarticular intervention (labral debridement) in conjunction with iliotibial band or psoas release. Four hips had a hip arthroscopy having not received it prior to the anteverting PAO. Review of the literature would therefore favor treatment of intraarticular pathology at the time of anteverting PAO, and the benefit of performing a femoral head–neck junction osteochondroplasty seems to be supported in the literature. The results of the present paper also show that performing an arthrotomy or arthroscopy with a major intraarticular intervention was associated with clinically meaningful improvement in PROM compared to anteverting PAO alone.

With regard to the technique used to visualize the joint, the findings of the present cohort demonstrate parity between arthrotomy and arthroscopic techniques in joint examination. This has previously been reported for patients undergoing PAO for classic hip dysplasia [28]. Arthroscopy is increasingly done today at the time of PAO to examine and treat the labrum and to perform a femoral head–neck junction osteochondroplasty, although some surgeons still perform an arthrotomy after acetabular correction to assess impingement and perform the osteochondroplasty. The current study would support either technique over anteverting PAO alone. Patients receiving either technique in conjunction with anteverting PAO demonstrated more consistent postoperative improvement in PROM compared to anteverting PAO alone, suggesting a benefit in joint visualization and treatment during index anteverting PAO.

Supporting the treatment of labral pathology at the time of surgery, the presence of labral lesions [29] or a detached labrum [30] has been associated with inferior outcomes or a risk of repeat hip arthroscopy, respectively, after PAO, suggesting that intraarticular procedures may positively influence labral health in excess of the mechanical offloading achieved through PAO alone. However, previous reports of 1 year PROM in patients receiving PAO with intraarticular procedures did not detect a differential effect of major versus minor interventions [28]. Longer follow-ups of 4.5 years in patients receiving PAO report that postoperative pain is not influenced by labral repair, excision or no treatment [8]. The results of the current cohort suggest that postoperative improvement in pain and function may be reliably achieved with major intraarticular procedures targeted at labral repair or relief of bony impingement on the labrum via femoral head–neck offset procedures when indicated. The small number of cases does not allow us to comment individually on labral repair versus head–neck junction osteochondroplasty, as the majority of patients underwent both procedures. Performing a major intraarticular intervention was not associated with an increased risk of reoperation, but equally important is the fact that treating the labrum or head–neck junction is not associated with a decreased risk of reoperation either. The fact is, despite having worse PROM scores, patients who did not have intraarticular intervention did not require further surgery to address the labrum or the head–neck junction. However, all patients in all groups had improvement in PROM, notably those who did not have intraarticular intervention failed to meet MCID in multiple PROM subsets, a difference that would not have not been seen when reviewing the data in aggregate.

Early reports of secondary anterior impingement after PAO underscore the importance of assessing acetabular version during fragment positioning [6, 23, 31]. The fact is that achieving appropriate acetabular version may be the most important predictor of PAO outcomes [30, 32]. Maintaining or improving range of motion at the time of PAO in patients with acetabular retroversion with or without underlying hip dysplasia is of equal importance. In the present study, patients who underwent head–neck junction osteochondroplasty had better postoperative PROM scores when compared to those who did not [2, 33]. So, similar to what has been presented for PAO in the setting of DDH, the data presented here supports performing a head–neck junction osteochondroplasty to address cam morphology after acetabular correction in order to relieve rim impingement further.

In conclusion, superior postoperative improvement in measures of pain and function was achieved with the execution of a major intraarticular intervention defined as a labral repair or head–neck junction osteochondroplasty. Surgeons performing anteverting PAO for acetabular retroversion should therefore perform open arthrotomy or arthroscopy to inspect and treat both labral pathology and femoral morphologic features to improve outcomes.

## SUPPLEMENTARY DATA

Supplementary data are available at *Journal of Hip Preservation Surgery* online.

## CONFLICTOF INTEREST STATEMENT

None declared.

## Data Availability

Data available on request.

## Supplementary Material

hnab040_Supplementary_Data

## References

[1] Ganz R, Klaue K, Vinh TS et al A new periacetabular osteotomy for the treatment of hip dysplasias. Technique and preliminary results. Clin Orthop Relat Res 1988; 232: 26–36. 3383491

[2] Zurmuhle CA, Anwander H, Albers CE et al Periacetabular osteotomy provides higher survivorship than rim trimming for acetabular retroversion. Clin Orthop Relat Res 2017; 475: 1138–50. 27921206 10.1007/s11999-016-5177-5 PMC5339145

[3] Ezoe M, Naito M, Inoue T. The prevalence of acetabular retroversion among various disorders of the hip. J Bone Joint Surg Am 2006; 88: 372–9. 16452750 10.2106/JBJS.D.02385

[4] Fujii M, Nakashima Y, Yamamoto T et al Acetabular retroversion in developmental dysplasia of the hip. J Bone Joint Surg Am 2010; 92: 895–903. 20360513 10.2106/JBJS.I.00046

[5] Ganz R, Leunig M, Leunig-Ganz K et al The etiology of osteoarthritis of the hip: an integrated mechanical concept. Clin Orthop Relat Res 2008; 466: 264–72. 18196405 10.1007/s11999-007-0060-z PMC2505145

[6] Siebenrock KA, Schoeniger R, Ganz R. Anterior femoro-acetabular impingement due to acetabular retroversion. Treatment with periacetabular osteotomy. J Bone Joint Surg Am 2003; 85: 278–86. 12571306 10.2106/00004623-200302000-00015

[7] Beck M, Kalhor M, Leunig M et al Hip morphology influences the pattern of damage to the acetabular cartilage: femoroacetabular impingement as a cause of early osteoarthritis of the hip. J Bone Joint Surg Br 2005; 87: 1012–8. 15972923 10.1302/0301-620X.87B7.15203

[8] Pitto RP, Klaue K, Ganz R. [Labrum lesions and acetabular dysplasia in adults]. Z Orthop Ihre Grenzgeb 1996; 134: 452–6. 8967147 10.1055/s-2008-1037437

[9] Wiberg G. Studies on dysplastic acetabula and congenital subluxation of the hip joint: with special reference to the complications of osteoarthritis. Acta Chir Scand 1939; 58: 7–38.

[10] Tonnis D, Heinecke A. Acetabular and femoral anteversion: relationship with osteoarthritis of the hip. J Bone Joint Surg Am 1999; 81: 1747–70. 10608388 10.2106/00004623-199912000-00014

[11] Lequesne M, de Seze J. False profile of the pelvis. A new radiographic incidence for the study of the hip. Its use in dysplasias and different coxopathies. Rev Rhum Mal Osteoartic 1961; 28: 643–52. 14464207

[12] Busse J, Gasteiger W, Tonnis D. A new method for roentgenologic evaluation of the hip joint—the hip factor. Arch Orthop Unfallchir 1972; 72: 1–9. 5020681 10.1007/BF00415854

[13] Harris WH. Traumatic arthritis of the hip after dislocation and acetabular fractures: treatment by mold arthroplasty. An end-result study using a new method of result evaluation. J Bone Joint Surg Am 1969; 51: 737–55. 5783851

[14] Biedermann R, Donnan L, Gabriel A et al Complications and patient satisfaction after periacetabular pelvic osteotomy. Int Orthop 2008; 32: 611–7. 17579861 10.1007/s00264-007-0372-3 PMC2551712

[15] Maranho DA, Williams KA, Millis MB et al Mid-term results of periacetabular osteotomy for the treatment of hip dysplasia associated with Down syndrome: minimum follow-up of five years. J Bone Joint Surg Am 2018; 100: 428–34. 29509620 10.2106/JBJS.17.00957

[16] Matheney T, Zaltz I, Kim Y-J et al Activity level and severity of dysplasia predict age at Bernese periacetabular osteotomy for symptomatic hip dysplasia. J Bone Joint Surg Am 2016; 98: 665–71. 27098325 10.2106/JBJS.15.00735

[17] McClincy MP, Wylie JD, Kim Y-J et al Periacetabular osteotomy improves pain and function in patients with lateral center-edge angle between 18 degrees and 25 degrees, but are these hips really borderline dysplastic? Clin Orthop Relat Res 2019; 477:1145–53. 30272611 10.1097/CORR.0000000000000516 PMC6494304

[18] Wasko MK, Yanik EL, Pascual-Garrido C et al Psychometric properties of patient-reported outcome measures for periacetabular osteotomy. J Bone Joint Surg Am 2019; 101: e21. 30893237 10.2106/JBJS.18.00185

[19] Nehme A, Trousdale R, Tannous Z et al Developmental dysplasia of the hip: is acetabular retroversion a crucial factor? Orthop Traumatol Surg Res 2009; 95: 511–9. 19837022 10.1016/j.otsr.2009.06.006

[20] Notzli HP, Wyss TF, Stoecklin CH et al The contour of the femoral head-neck junction as a predictor for the risk of anterior impingement. J Bone Joint Surg Br 2002; 84: 556–60. 12043778 10.1302/0301-620x.84b4.12014

[21] Benjamini Y, Hochberg Y. Controlling the false discovery rate—a practical and powerful approach to multiple testing. J R Stat Soc Ser B Stat Methodol 1995; 57: 289–300.

[22] Belzile EL, Beaulé PE, Ryu JJ et al Outcomes of joint preservation surgery: comparison of patients with developmental dysplasia of the hip and femoroacetabular impingement. J Hip Preserv Surg 2016; 3: 270–7. 29632687 10.1093/jhps/hnw033 PMC5883182

[23] Reynolds D, Lucas J, Klaue K. Retroversion of the acetabulum. A cause of hip pain. J Bone Joint Surg Br 1999; 81: 281–8. 10204935 10.1302/0301-620x.81b2.8291

[24] Siebenrock KA, Schaller C, Tannast M et al Anteverting periacetabular osteotomy for symptomatic acetabular retroversion results at ten years. J Bone Joint Surg Am 2014; 96:1785–92. 25378505 10.2106/JBJS.M.00842

[25] Peters CL, Anderson LA, Erickson JA et al An algorithmic approach to surgical decision making in acetabular retroversion. Orthopedics 2011; 34: 10. 21210626 10.3928/01477447-20101123-07 PMC3399593

[26] Parry JA, Swann RP, Erickson JA et al Midterm outcomes of reverse (anteverting) periacetabular osteotomy in patients with hip impingement secondary to acetabular retroversion. Am J Sports Med 2016; 44: 672–6. 26712890 10.1177/0363546515620382

[27] Wyatt MC, Smith C, Zavareh A et al Functional acetabular retroversion syndrome: description of a specific sub-type of FAI and results of treatment with minimally invasive PAO. Hip Int 2019; 30: 779–86. 31177851 10.1177/1120700019855240

[28] Wyles CC, Hevesi M, Bartels DW et al Arthroscopy and arthrotomy to address intra-articular pathology during PAO for hip dysplasia demonstrates similar short-term outcomes. J Hip Preserv Surg 2018; 5: 282–95. 30393556 10.1093/jhps/hny022 PMC6206691

[29] Siebenrock KA, Schöll E, Lottenbach M et al Bernese periacetabular osteotomy. Clin Orthop Relat Res 1999; 363: 9–20. 10379300

[30] Hartig-Andreasen C, Troelsen A, Thillemann TM et al Risk factors for the need of hip arthroscopy following periacetabular osteotomy. J Hip Preserv Surg 2015; 2: 374–84. 27011862 10.1093/jhps/hnv053 PMC4732374

[31] Myers SR, Eijer H, Ganz R. Anterior femoroacetabular impingement after periacetabular osteotomy. Clin Orthop Relat Res 1999; 363: 93–9. 10379309

[32] Albers CE, Steppacher SD, Ganz R et al Impingement adversely affects 10-year survivorship after periacetabular osteotomy for DDH. Clin Orthop Relat Res 2013; 471: 1602–14. 23354462 10.1007/s11999-013-2799-8 PMC3613512

[33] Albers CE, Steppacher SD, Tannast M et al Surgical technique: reverse periacetabular osteotomy. In: Nho S, Leunig M, Kelly B et al (eds). Hip Arthroscopy and Hip Joint Preservation Surgery. New York, NY: Springer New York, 2013, 1–17.

