# Supplementary material for: Addressing intraarticular pathology at the time of anteverting periacetabular osteotomy for acetabular retroversion is associated with better short-term patient-reported outcomes
Source: J Hip Preserv Surg. 2021 Jun 20;8(1):90–104. doi: 10.1093/jhps/hnab040 (PMC8527802; doi:10.1093/jhps/hnab040)
Supplement: hnab040_Supplementary_Data [file hnab040_supplementary_data.docx]

**SUPPLEMENTARY DATA**

**Supplementary Table 1.** Pre- and post-operative patient-reported outcome measures, by surgical technique performed and preoperative diagnosis.

|  | Diagnosis of DDH + Retroversion | | | Diagnosis of Retroversion | | |
| --- | --- | --- | --- | --- | --- | --- |
|  | Ante. PAO (N=3) | Ante. PAO +  Arthrotomy (N=10) | Ante. PAO +  Arthroscopy (N=10) | Ante. PAO (N=5) | Ante. PAO +  Arthrotomy (N=13) | Ante. PAO +  Arthroscopy (N=7) |
| **UCLA Score** |  |  |  |  |  |  |
| Preoperative | 7.5 (3.5) | 7.4 (2.3) | 5.4 (2.9) | 7.0 (2.1) | 7.3 (2.4) | 7.6 (2.6) |
| Postoperative | 7.0 (2.0) | 6.0 (2.5) | 7.1 (2.0) | 8.6 (1.9) | 7.9 (2.1) | 6.8 (2.6) |
| Change (Post-Pre) | -0.5 (0.7) | -1.3 (3.5) | 1.6 (1.3) | 1.6 (2.8) | 0.4 (2.1) | -1.3 (2.5) |
|  |  |  |  |  |  |  |
| **Harris Hip Score** |  |  |  |  |  |  |
| Preoperative | 52.8 (34.2) | 59.6 (10.0) | 54.5 (21.2) | 64.2 (15.0) | 61.1 (20.0) | 58.9 (24.1) |
| Postoperative | 73.3 (28.1) | 74.7 (22.5) | 83.6 (15.7) | 98.3 (2.4) | 91.2 (13.0) | 73.1 (25.6) |
| Change (Post-Pre) | 9.4 (5.4) | 16.9 (20.5) | 30.5 (12.6) | 34.1 (16.2) | 33.6 (20.8) | 11.0 (44.4) |
|  |  |  |  |  |  |  |
| **HOOS Total Pain** |  |  |  |  |  |  |
| Preoperative | 38.8 (26.5) | 52.3 (12.5) | 53.4 (23.4) | 66.5 (23.2) | 61.3 (22.5) | 61.8 (15.9) |
| Postoperative | 73.3 (31.7) | 75.3 (27.0) | 82.8 (20.6) | 97.5 (3.5) | 91.3 (15.0) | 70.8 (24.6) |
| Change (Post-Pre) | 22.5 (7.1) | 22.8 (19.8) | 33.5 (10.1) | 38.1 (18.8) | 33.8 (18.6) | 8.8 (22.7) |
|  |  |  |  |  |  |  |
| **HOOS Total ADL** |  |  |  |  |  |  |
| Preoperative | 51.5 (27.0) | 61.6 (13.0) | 60.5 (25.4) | 80.9 (17.9) | 77.7 (20.4) | 77.1 (16.8) |
| Postoperative | 77.5 (32.9) | 84.6 (20.0) | 88.2 (16.5) | 99.3 (1.5) | 94.9 (7.8) | 86.1 (11.2) |
| Change (Post-Pre) | 14.7 (10.4) | 21.3 (16.9) | 32.4 (14.5) | 23.2 (17.0) | 20.9 (20.6) | 7.4 (21.4) |
|  |  |  |  |  |  |  |
| **HOOS Total S&R** |  |  |  |  |  |  |
| Preoperative | 34.4 (13.3) | 39.4 (14.7) | 32.0 (17.8) | 57.5 (27.0) | 48.6 (26.2) | 51.8 (21.6) |
| Postoperative | 52.1 (41.6) | 60.2 (37.9) | 74.2 (29.6) | 97.5 (5.6) | 86.4 (17.4) | 64.6 (29.5) |
| Change (Post-Pre) | 0.0 (26.5) | 16.4 (37.0) | 51.8 (19.3) | 40.0 (26.0) | 40.3 (25.8) | 14.6 (30.0) |
|  |  |  |  |  |  |  |
| **HOOS Total QoL** |  |  |  |  |  |  |
| Preoperative | 40.6 (13.3) | 25.0 (12.5) | 20.3 (14.1) | 42.5 (19.5) | 29.8 (22.3) | 43.8 (21.7) |
| Postoperative | 52.1 (40.2) | 57.0 (28.0) | 63.3 (24.9) | 88.8 (12.8) | 79.5 (23.4) | 58.3 (27.3) |
| Change (Post-Pre) | 3.1 (39.8) | 32.0 (27.8) | 50.9 (21.2) | 46.3 (30.8) | 50.0 (22.9) | 14.6 (34.8) |
|  |  |  |  |  |  |  |
| **WOMAC Total Pain** |  |  |  |  |  |  |
| Preoperative | 45.0 (28.3) | 57.5 (13.2) | 61.3 (27.2) | 73.0 (23.3) | 68.8 (23.9) | 67.9 (17.3) |
| Postoperative | 80.0 (26.5) | 81.3 (23.1) | 87.5 (17.9) | 99.0 (2.2) | 92.3 (15.7) | 74.2 (27.8) |
| Change (Post-Pre) | 25.0 (0.0) | 23.8 (21.2) | 30.0 (17.8) | 26.0 (23.0) | 27.3 (19.0) | 6.7 (20.9) |
|  |  |  |  |  |  |  |
| **WOMAC Total Stiffness** |  |  |  |  |  |  |
| Preoperative | 56.3 (26.5) | 47.5 (22.7) | 39.1 (24.5) | 60.0 (27.1) | 64.4 (19.7) | 66.1 (24.7) |
| Postoperative | 79.2 (19.1) | 59.4 (27.3) | 71.9 (17.4) | 97.5 (5.6) | 89.8 (16.6) | 82.1 (18.9) |
| Change (Post-Pre) | 12.5 (17.7) | 12.5 (28.3) | 33.9 (20.0) | 37.5 (29.3) | 25.0 (16.8) | 16.1 (24.7) |
|  |  |  |  |  |  |  |
| **WOMAC Total Physical** |  |  |  |  |  |  |
| Preoperative | 51.5 (27.0) | 61.6 (13.0) | 60.5 (25.4) | 80.9 (17.9) | 77.7 (20.4) | 77.1 (16.8) |
| Postoperative | 77.5 (32.9) | 84.6 (20.0) | 88.2 (16.5) | 99.4 (1.3) | 94.9 (7.8) | 88.1 (11.5) |
| Change (Post-Pre) | 14.7 (10.4) | 21.3 (16.9) | 32.4 (14.5) | 18.5 (18.0) | 20.9 (20.6) | 11.0 (21.7) |
|  |  |  |  |  |  |  |
| **WOMAC Total TOTAL** |  |  |  |  |  |  |
| Preoperative | 50.5 (27.3) | 59.6 (12.2) | 58.9 (25.1) | 77.5 (18.9) | 75.2 (20.3) | 74.3 (16.4) |
| Postoperative | 78.1 (30.1) | 81.8 (20.6) | 86.7 (16.0) | 99.2 (1.9) | 93.9 (9.6) | 83.0 (14.3) |
| Change (Post-Pre) | 16.7 (5.9) | 21.1 (16.8) | 32.0 (14.5) | 21.7 (19.1) | 22.3 (19.3) | 7.7 (20.0) |
|  |  |  |  |  |  |  |
| **SF12 Physical** |  |  |  |  |  |  |
| Preoperative | 28.6 (15.5) | 34.3 (8.5) | 34.5 (9.6) | 46.2 (2.8) | 39.2 (12.0) | 36.4 (12.4) |
| Postoperative | 48.2 (12.8) | 44.9 (12.0) | 50.8 (10.0) | 56.4 (0.8) | 52.5 (8.0) | 43.7 (16.2) |
| Change (Post-Pre) | 13.6 (5.1) | 10.0 (12.4) | 16.2 (8.2) | 10.2 (2.1) | 14.9 (9.6) | 7.3 (18.7) |
|  |  |  |  |  |  |  |
| **SF12 Mental** |  |  |  |  |  |  |
| Preoperative | 66.5 (4.6) | 45.4 (12.0) | 50.9 (11.5) | 55.7 (7.9) | 53.9 (12.2) | 54.8 (9.3) |
| Postoperative | 59.0 (8.7) | 49.1 (11.5) | 52.3 (5.4) | 55.5 (3.6) | 51.1 (11.5) | 52.7 (12.1) |
| Change (Post-Pre) | -2.5 (3.2) | 3.6 (17.1) | -0.2 (15.2) | -0.2 (4.5) | -3.9 (11.1) | -2.1 (10.8) |

**Supplementary Table 2.** Pre- and post-operative patient-reported outcome measures, by intraarticular intervention and preoperative diagnosis

|  | Diagnosis of DDH + Retroversion | | | Diagnosis of Retroversion | | | |
| --- | --- | --- | --- | --- | --- | --- | --- |
|  | No Intervention (N=8) | Minor (N=1) | Major (N=14) | No Intervention (N=6) | | Minor (N=6) | Major (N=13) |
| **UCLA Score** |  |  |  |  |  | |  |
| Preoperative | 8.1 (2.0) | 10.0 ( ) | 5.4 (2.6) | 6.8 (1.9) | 7.3 (2.8) | | 7.5 (2.4) |
| Postoperative | 6.4 (2.4) | 9.0 ( ) | 6.5 (2.1) | 8.2 (2.0) | 5.8 (1.5) | | 8.3 (2.2) |
| Change (Post-Pre) | -1.8 (3.7) | -1.0 ( ) | 1.2 (1.5) | 1.3 (2.6) | -2.0 (2.4) | | 0.4 (2.2) |
|  |  |  |  |  |  | |  |
| **Harris Hip Score** |  |  |  |  |  | |  |
| Preoperative | 58.3 (16.4) | 67.1 ( ) | 55.2 (18.1) | 63.8 (13.5) | 58.7 (9.7) | | 60.9 (24.7) |
| Postoperative | 73.2 (24.8) | 81.4 ( ) | 81.1 (18.2) | 91.9 (16.0) | 74.8 (21.8) | | 90.1 (18.2) |
| Change (Post-Pre) | 12.7 (22.3) | 14.3 ( ) | 27.7 (13.1) | 28.1 (20.7) | 14.6 (18.9) | | 31.6 (34.9) |
|  |  |  |  |  |  | |  |
| **HOOS Total Pain** |  |  |  |  |  | |  |
| Preoperative | 48.9 (15.7) | 67.5 ( ) | 51.5 (20.3) | 61.3 (24.4) | 63.3 (15.2) | | 62.7 (21.8) |
| Postoperative | 70.7 (27.8) | 87.5 ( ) | 82.0 (22.6) | 88.0 (21.5) | 68.9 (26.0) | | 94.1 (8.9) |
| Change (Post-Pre) | 17.5 (18.2) | 20.0 ( ) | 33.7 (11.5) | 33.5 (19.3) | 6.4 (13.9) | | 34.3 (22.2) |
|  |  |  |  |  |  | |  |
| **HOOS Total ADL** |  |  |  |  |  | |  |
| Preoperative | 54.8 (13.8) | 85.3 ( ) | 61.2 (21.3) | 78.7 (16.9) | 80.9 (12.0) | | 76.5 (22.3) |
| Postoperative | 77.3 (26.5) | 100.0 ( ) | 88.5 (14.7) | 95.9 (7.7) | 83.3 (10.0) | | 96.5 (6.7) |
| Change (Post-Pre) | 17.6 (19.8) | 14.7 ( ) | 30.6 (12.3) | 21.5 (15.2) | 1.3 (10.9) | | 23.5 (23.1) |
|  |  |  |  |  |  | |  |
| **HOOS Total S&R** |  |  |  |  |  | |  |
| Preoperative | 35.7 (14.3) | 56.3 ( ) | 34.4 (16.3) | 54.2 (25.5) | 41.7 (21.9) | | 54.3 (25.6) |
| Postoperative | 51.8 (38.0) | 87.5 ( ) | 71.0 (32.0) | 89.6 (20.0) | 63.8 (29.8) | | 88.1 (16.6) |
| Change (Post-Pre) | 8.3 (36.8) | 31.3 ( ) | 41.3 (30.4) | 35.4 (25.8) | 21.3 (28.2) | | 37.5 (30.5) |
|  |  |  |  |  |  | |  |
| **HOOS Total QoL** |  |  |  |  |  | |  |
| Preoperative | 33.9 (12.4) | 31.3 ( ) | 18.8 (12.2) | 39.6 (18.8) | 28.1 (22.3) | | 38.5 (23.5) |
| Postoperative | 56.3 (26.3) | 62.5 ( ) | 60.2 (30.3) | 81.3 (21.7) | 51.3 (21.8) | | 84.1 (21.2) |
| Change (Post-Pre) | 21.9 (30.3) | 31.3 ( ) | 45.6 (27.5) | 41.7 (29.8) | 17.5 (31.4) | | 48.3 (29.3) |
|  |  |  |  |  |  | |  |
| **WOMAC Total Pain** |  |  |  |  |  | |  |
| Preoperative | 55.0 (16.1) | 70.0 ( ) | 58.3 (23.8) | 67.5 (24.8) | 70.0 (17.9) | | 70.0 (22.7) |
| Postoperative | 77.1 (25.1) | 90.0 ( ) | 87.3 (18.2) | 90.8 (20.1) | 72.0 (30.5) | | 95.5 (7.9) |
| Change (Post-Pre) | 17.5 (20.2) | 20.0 ( ) | 32.5 (15.9) | 23.3 (21.6) | 4.0 (13.9) | | 28.2 (21.4) |
|  |  |  |  |  |  | |  |
| **WOMAC Total Stiffness** |  |  |  |  |  | |  |
| Preoperative | 50.0 (20.4) | 75.0 ( ) | 39.6 (23.7) | 60.4 (24.3) | 60.4 (24.3) | | 67.3 (20.8) |
| Postoperative | 60.7 (23.3) | 75.0 ( ) | 71.6 (23.1) | 89.6 (20.0) | 80.0 (20.9) | | 92.7 (11.3) |
| Change (Post-Pre) | 2.1 (20.0) | 0.0 ( ) | 35.0 (20.2) | 29.2 (33.2) | 15.0 (10.5) | | 27.1 (20.5) |
|  |  |  |  |  |  | |  |
| **WOMAC Total Physical** |  |  |  |  |  | |  |
| Preoperative | 54.8 (13.8) | 85.3 ( ) | 61.2 (21.3) | 78.7 (16.9) | 80.9 (12.0) | | 76.5 (22.3) |
| Postoperative | 77.3 (26.5) | 100.0 ( ) | 88.5 (14.7) | 96.6 (7.1) | 83.3 (10.0) | | 96.8 (6.5) |
| Change (Post-Pre) | 17.6 (19.8) | 14.7 ( ) | 30.6 (12.3) | 17.9 (16.2) | 1.3 (10.9) | | 24.4 (21.9) |
|  |  |  |  |  |  | |  |
| **WOMAC Total TOTAL** |  |  |  |  |  | |  |
| Preoperative | 54.5 (13.8) | 81.3 ( ) | 58.8 (21.0) | 74.8 (18.2) | 76.9 (12.9) | | 74.9 (21.7) |
| Postoperative | 75.9 (25.2) | 95.8 ( ) | 86.8 (15.1) | 94.8 (10.8) | 80.7 (14.1) | | 95.9 (7.1) |
| Change (Post-Pre) | 16.3 (18.0) | 14.6 ( ) | 31.4 (11.9) | 20.0 (17.6) | 3.0 (7.5) | | 24.5 (22.4) |
|  |  |  |  |  |  | |  |
| **SF12 Physical** |  |  |  |  |  | |  |
| Preoperative | 31.4 (8.6) | 46.2 ( ) | 34.2 (9.3) | 44.0 (5.9) | 35.5 (8.7) | | 39.9 (13.5) |
| Postoperative | 44.0 (11.9) | 57.8 ( ) | 49.5 (10.4) | 54.7 (4.3) | 40.0 (18.3) | | 53.1 (7.4) |
| Change (Post-Pre) | 9.6 (13.1) | 11.6 ( ) | 15.1 (8.6) | 10.7 (2.2) | 4.0 (17.4) | | 15.1 (12.2) |
|  |  |  |  |  |  | |  |
| **SF12 Mental** |  |  |  |  |  | |  |
| Preoperative | 45.7 (16.7) | 58.4 ( ) | 51.3 (10.1) | 57.9 (8.9) | 60.4 (3.3) | | 50.3 (11.5) |
| Postoperative | 52.9 (10.3) | 57.1 ( ) | 50.9 (9.4) | 56.5 (4.0) | 52.8 (8.6) | | 50.5 (12.9) |
| Change (Post-Pre) | 8.3 (13.5) | -1.3 ( ) | -2.7 (15.4) | -1.4 (5.1) | -7.0 (9.1) | | -1.2 (11.6) |
